# Investigation of protective effects of olanzapine on impaired learning and memory using behavioral tests in a rat model of Alzheimer’s disease

**DOI:** 10.3389/fnagi.2025.1376074

**Published:** 2025-02-13

**Authors:** Somayeh Komaki, Parsa Amiri, Samaneh Safari, Ebrahim Abbasi, Fatemeh Ramezani-Aliakbari, Mandana Golipoor, Masoumeh Kourosh-Arami, Masome Rashno, Alireza Komaki

**Affiliations:** ^1^Department of Neuroscience, School of Science and Advanced Technologies in Medicine, Hamadan University of Medical Sciences, Hamadan, Iran; ^2^Department of Biochemistry, School of Medicine, Hamadan University of Medical Sciences, Hamadan, Iran; ^3^Department of Physiology, School of Medicine, Hamadan University of Medical Sciences, Hamadan, Iran; ^4^Neuroscience Research Center, Guilan University of Medical Sciences, Rasht, Iran; ^5^Asadabad School of Medical Sciences, Asadabad, Iran

**Keywords:** Alzheimer’s disease, Aβ, olanzapine, cognition, anxiety-like behavior, rat

## Abstract

**Introduction:**

Evidence suggests that oxidative stress plays a critical role in the pathogenesis and progression of Alzheimer’s disease (AD). Consequently, antioxidants may mitigate neurotoxicity induced by beta-amyloid (Aβ) and potentially reduce cell death. Previous research has demonstrated that olanzapine (OLZ) possesses antioxidant and neuroprotective properties. In this study, we investigated the protective and therapeutic effects of OLZ on an animal model of AD induced by Aβ using behavioral assessments.

**Methods:**

Rats were randomly assigned to one of five groups (*n* = 10 rats per group): a control group, a sham group that received an intracerebrovascular (ICV) injection of phosphate-buffered saline (the solvent for Aβ), an AD group that received an ICV injection of Aβ, an OLZ group that received OLZ *via* gavage for two months, and an AD + OLZ group that received OLZ for one month before and one month after AD induction.

**Results:**

We used the Elevated Plus Maze (EPM), Novel Object Recognition Test (NORT), Barnes Maze (BM), Passive Avoidance Test (PAT), and Morris Water Maze (MWM) to assess behavioral performance in the experimental rats. Aβ administration impaired cognition and increased anxiety-like behavior. Treatment with OLZ improved cognitive decline and reduced anxiety-like behavior in Aβ-infused rats.

**Conclusion:**

Our findings suggest that OLZ can restore cognitive performance and alleviate anxiety-like behavior following Aβ injection. Thus, OLZ may have both preventive and therapeutic potential for AD and could be considered a viable pharmacological option.

## Introduction

Alzheimer’s disease (AD) is the most common type of memory impairment that leads to damage to the nervous system (Zhu et al., 2013). AD is the sixth leading cause of death and the fifth leading cause of death in people over the age of 65 (Reitz, 2012). A study of the prevalence of AD in the United States showed that the number of older people with AD increased from about 4.7 million in 2010 to almost three times in 2015, which will also face an increasing trend by 2025 because the population of the elderly is increasing (Weuve et al., 2015). AD is a chronic neurodegenerative disease that slowly causes irreversible damage to neurons, resulting in a gradual decline in cognitive and memory abilities (Zhu et al., 2013). The disease worsens over time and is the cause of 60–70% of dementia. The most common symptom of the disease is difficulty in remembering recent events (short-term memory loss). As the disease progresses, the symptoms include language problems, impaired awareness of the three dimensions of time and place and person, mood swings, loss of motivation, inability to take care of yourself, and behavioral issues (Brown et al., 2009). Although the pathological and clinical signs of AD have been identified, the exact cause remains unknown, and this uncertainty hinders the effectiveness of preventive or curative processes for the disease (Reinke and Gestwicki, 2011). At the pathological level, patients suffer from beta-amyloid (Aβ) plaque deposition, disrupted formation of neurofibrillary tangles, synaptic dysfunction in areas involved in learning and memory, and disruption in other cognitive functions in the brain. To date, loss of synapses and neuronal death have been identified as responsible for most AD symptoms (Terry et al., 1991). There are currently several competing hypotheses to explain the cause of this disease, including genetic and cholinergic factors, Aβ, Tau, and oxidative stress (Behl, 1999; Butterfield et al., 2007; Liu et al., 2019; Pohanka, 2014).

The central nervous system (CNS) is highly susceptible to oxidative damage due to its high amount of unsaturated fatty acids. In contrast, its antioxidant system is weaker than other organs (Reddy et al., 2010). Oxidative stress markers can be seen even earlier than pathological changes in AD, and Aβ peptide seems to be the main factor in the formation of these markers (Butterfield and Boyd-Kimball, 2004). Tissue culture experiments and initial studies have shown that Aβ is highly toxic to neurons and can cause complete neuronal death after exposure of the cells to it for 24 h. Evidence suggests that neuronal death is due to apoptosis and the oxidative effects of Aβ. Evidence suggests that intraneuronal Aβ can enter the mitochondria, disrupt the electron transport chain, and produce reactive oxygen species (ROS), including hydroxyl (OH) superoxide (O_2_), and hydrogen peroxide (H_2_O_2_) radicals (Grundke-Iqbal et al., 1986; O’brien and Wong, 2011; Praticò et al., 2002; Wang et al., 2013). Chronic exposure to this peptide causes the release of chemokines and some harmful cytokines, such as interleukin (IL)-1 beta, IL-6, IL-8, tumor necrosis factor-alpha (TNF-α), and the inflammatory protein macrophage 1 alpha (Murgas et al., 2012). In this regard, it has been reported that IL-1 alpha and IL-1 beta are important proinflammatory cytokines in the brain of AD patients (Dursun et al., 2015).

ROS and reactive nitrogen species (RNS) via lipid peroxidation of cell membranes and organelle membranes produce hydroxynonal and malondialdehyde (MDA) toxins (Perry et al., 2011). Lipid peroxidation of the membrane also promotes the phosphorylation of tau protein and its accumulation to form neurofibrillary tangles (Avila, 2006). Oxidative stress has been shown to play an important role in cognitive disorders, such as AD (Grundke-Iqbal et al., 1986; O’brien and Wong, 2011; Praticò et al., 2002; Wang et al., 2013). Thus, antioxidants may reduce neurotoxicity induced by Aβ and reduce cell death, leading to improved cognitive impairment and memory due to AD (Aliev et al., 2008). Increased sensitivity to oxidative stress and inflammation in the elderly brain may lead to cognitive impairment; however, daily intake of antioxidants and anti-inflammatory agents can greatly delay oxidative damage to nerve tissue and may have a tremendous impact (Johnson, 2012).

**FIGURE 1:**
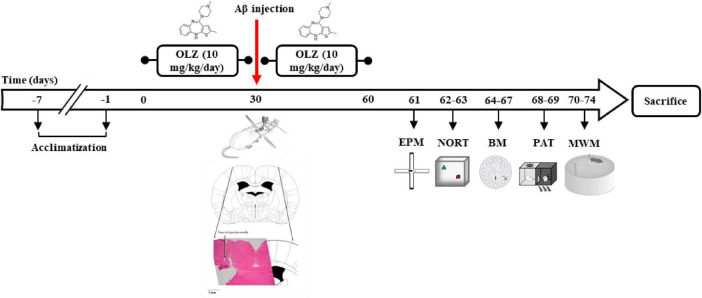
The experimental timeline. Following 30 days of olanzapine administration (OLZ; 10 mg/kg) in experimental groups, to generate a rat model of Alzheimer’s disease (AD), the rats were anesthetized and ICV injection of 5 μL Aβ was conducted. Afterward, OLZ was re-administered by daily oral gavage for 30 days. The animals’ behavior was then investigated using an elevated plus maze (EPM), novel object recognition test (NORT), Barnes maze (BM), passive avoidance task (PAT), and Morris water maze (MWM).

Olanzapine (OLZ) is one of the new atypical antipsychotic drugs (including risperidone, quetiapine, sertindole, and OLZ) and one of the drugs of the thienobenzodiazepine group (Figure 1). It is effective in treating schizophrenia, psychosis, and anxiety. The mechanism of its antipsychotic effect is due to its antagonistic properties on serotonin 5-HT2A and 5-HT2C receptors as well as dopamine D1-D4 receptors and the inhibition of the reuptake of serotonin and dopamine at the presynaptic end of CNS nerves. In addition, it has a slight affinity for GABA-A and adrenergic receptors and inhibits them. Anticholinergic properties of this drug have also been observed. OLZ binds to muscarinic (M1–M5) and histamine (H1) receptors, as well (Johari et al., 2013; Leinonen et al., 2005).

OLZ has antioxidant properties and is used to treat patients with schizophrenia. Treatment with OLZ for 2 months significantly (37%) increased total antioxidant capacity (TAC) and decreased MDA (22.2%), indicating its antioxidant properties (70). OLZ has also been shown to have direct effects on intracellular mechanisms that affect cell death through apoptosis. OLZ increases the expression of superoxide dismutase (SOD) and decreases the expression of neurotrophin p75 in PC12 cells. Increasing SOD and decreasing p75 can reduce cell death in cell culture systems (Csernansky et al., 2006). In vitro, OLZ exerts its antioxidant effects by modifying O_2_. Thus, patients with schizophrenia treated with OLZ were at lower risk for free radical damage (Dietrich-Muszalska et al., 2013; Singh et al., 2008). Treatment of psychiatric patients with OLZ in the first episode of the disease for 24 weeks increased SOD activity to normal values. Evans et al. also showed that in patients with schizophrenia treated with atypical antipsychotic drugs, such as risperidone, clozapine (CLZ), and OLZ, plasma levels of antioxidant enzymes improved and plasma levels of lipid peroxide decreased (Evans et al., 2003). In this regard, Brinholi et al. (2016) reported that CLZ and OLZ are more effective antioxidants than haloperidol (HAL), quetiapine, risperidone, and ziprasidone *in vitro*.

OLZ exhibits a range of effects on anxiety, learning, and memory, which appear to depend on factors such as treatment duration, sex, and experimental conditions. Studies demonstrate its anxiolytic properties in both clinical and preclinical settings. For instance, patients receiving OLZ showed significant improvements in anxiety ratings compared to bromazepam (Moretti et al., 2004), and preclinical studies have shown its intrinsic anxiolytic properties independent of its antipsychotic effects or influence on motor functions (Mead et al., 2008). However, findings in the open field and light/dark box tests indicate a sex-dependent impact, with decreased anxiety in males and increased anxiety in females (Lockington and Hughes, 2021). Additionally, the anxiolytic effect was observed acutely in stressed rats but diminished with chronic treatment (Locchi et al., 2008). Regarding learning and memory, results are somewhat mixed. OLZ impaired performance in the Morris water maze in naïve mice (MutluO, 2011), while other studies reported no significant effect on acquisition, consolidation, or retrieval in the step-through test (Hou et al., 2006). Interestingly, OLZ improved spatial learning in the Morris water maze task and showed superior performance in Barnes maze trials compared to lipopolysaccharide-treated groups (Tachi et al., 2021). These findings suggest that while OLZ may generally improve spatial learning and possess anxiolytic effects, its impact is nuanced and influenced by treatment length, sex, and baseline stress conditions.

OLZ also has neuroprotective properties (Brinholi et al., 2016; Dakhale et al., 2004; He et al., 2004; Onder et al., 2013). On the other hand, studies have shown conflicting results regarding the effect of OLZ on memory impairment and AD (Kennedy et al., 2005; Kennedy et al., 2001; Larson and McCurry, 2007; Rankin and Layne, 2005; Schatz, 2003). However, there is still debate whether OLZ can be used as a preventative or therapeutic agent for neurodegenerative diseases, including AD. In this study, OLZ was considered as a protective compound (neuroprotective and anti-neuroinflammatory agent, oxidative stress reducer, and neurogenesis stimulant) against AD, and it was tried to find whether this compound can have protective effects on neurons in AD rats or can prevent memory and learning disorders in AD rats. Therefore, the practical purpose of this study was to investigate the neuroprotective role of OLZ in AD and find a cost-effective treatment with fewer side effects.

## Materials and methods

### Animals

In this experimental study, 50 male Wistar rats weighing 250–280 g were obtained from the animal house of the Hamadan University of Medical Sciences. Sufficient water and food were provided to the rats during the experiments. A temperature of 20 ± 2°C, a humidity of 50–60%, and a 12:12-h light: dark cycle (from 7 a.m. to 7 p.m.) were considered. For 1 week, the animals were handled daily to avoid the stress of catching and working with them during the test. The treatment procedures and the protocols of animal health surveillance were by the Veterinary Ethics Committee of the Hamadan University of Medical Sciences, based on the National Institutes of Health Guidelines for studies involving animals (NIH Publication 80–23, 1996).

### Experimental design

After acclimatizing the rats to the environment, we randomly divided the animals into the following groups:

Control group: normal control rats.

Sham group: This group underwent surgery and received the solvent of Aβ instead of Aβ (ICV injection of 5 μl phosphate-buffered saline).

OLZ (Sigma-Aldrich, European Pharmacopoeia) group: This group received OLZ at a dose of 10 mg/kg orally by gavage (2 months) without surgery.

AD group: This group underwent surgery and received Aβ (ICV injection of 5 μL Aβ) for the induction of AD.

AD + OLZ group: This group underwent surgery and received Aβ (ICV injection of 5 μL Aβ) and OLZ 1 month before surgery and 1 month after surgery.

OLZ was dissolved in normal saline as a vehicle, immediately before use from 9 to 10 am. All behavioral tests were performed between 10 and 12 a.m. The experimental timeline is shown in Figure 1.

### Aβ injections and induction of Alzheimer’s model

Before using of Aβ_1–42_ (Tocris Bioscience, Bristol, United Kingdom), it was dissolved in 100 μL of PBS and incubated at 37°C for 7 days. For AD induction, the animals were first anesthetized by intraperitoneal injection of ketamine (100 mg/kg) and xylazine (10 mg/kg). After anesthesia, the animals were placed in a stereotaxic device and the ear bar of the stereotaxic device was placed inside the animal’s ear, and after observing the ocular reflex, the head was fixed in the device. The scalp was shaved and the surface of the skull was exposed. Aβ_1–42_ (5 μL) was injected unilaterally into the brain by stereotaxic surgery using Paxinos and Watson atlas (the coordinates of the appendix: 2 mm lateral to the midline, 1.2 mm posterior to bregma, and 4 mm ventral to the surface of the cortex) through a Hamilton syringe in the cerebroventricular region (Azadeh et al., 2016; Figure 1). To prevent brain damage, the injection was given slowly for 20 min. For better deposition of Aβ, the Aβ administration was performed in three 5-min periods with 2-min breaks between them. After the ICV injection, each animal spent some time in individual cages, then after return to the home cage. It takes 2 weeks to develop an AD model (Ahmadi et al., 2021; Asadbegi et al., 2018).

### Evaluation of behavioral functions

#### Elevated plus maze

To evaluate the animal’s anxiety-like behavior, EPM is used. The device is a maze made of wood, which is 50 cm away from the floor and consists of two open and two closed arms that are facing each other. The maze has two black open arms (with no walls) with dimensions of 30 × 10 cm and two closed arms with dimensions of 30 × 15 × 10 cm (with a wall) black. For 10 min before the experiment, each animal was placed on an elevated plate in an unfamiliar environment to reduce accidental errors in entering the arms. At the beginning of the experiment, the animal was placed in the center of the maze, with the direction of the animal’s head toward the close arms. Entry into each arm is when the animal has all four paws in the arm (94). The test lasts for 10 min and is done only once for each animal. After each experiment, the maze was cleaned with 70% ethanol alcohol. The EPM was also located under a CCTV camera, and the animal’s behavior in the maze was transmitted to the computer by a camera and saved to analyze the data.

Rats tend to be curious about the new environment and maze arms when they are located in the maze. The animal explores the new environment or escapes from the new environment. Due to the height of the maze from the ground, the shape of the open arms, the fear, and anxiety of falling from these arms, rats are less likely to enter and remain in the open arms. In addition, in the EPM, the animals actively avoid open arms. Thus, the open arm entrance is a measure of anxiety, while entry into the closed arm is an indicator of the animal’s general activity. Known anti-anxiety agents are able to increase the tendency of an animal to enter and remain in open arms (Clement and Chapouthier, 1998). At the end of each test, maze was cleaned with 70% ethanol to eliminate olfactory cues.

#### Novel object recognition test

It is a suitable model for examining the tendency of rodents to look for new objects in comparison with old objects that they are familiar with, which also examines diagnostic memory and the rate of searching behavior in rodents. The test has steps, including adaption to an empty container, getting acquainted with two identical objects on the 1st day, and the testing step on the 2nd day. The time elapsed to detect each object (object exploration time) and the time elapsed next to the new object (new object exploration time) are measured in this test. NORT is a simple learning test with the least motivational components that use rats’ innate tendency to spend more time exploring newer objects than more familiar ones. It is a benchmark for cognitive memory in rodents and works based on hippocampal integrity (Winocur and Greenwood, 1999).

In this study, large cubes (due to their high weight that cannot be moved when rats are examined), of the same material and free of any rust (because rust could be a visual sign for rats) were used as objects. The cubes were placed inside a wooden chamber with a length and width of about 40 × 50 cm and a height of 40 cm with a dull color (to isolate the inside of the chamber from the outside and reduce the impact of environmental factors). The light of the chamber was also adjusted so that no shadow was created. Objects were thoroughly washed between the steps to eliminate olfactory symptoms. The time spent next to each object was measured by a timer. Animals were and trained tested in three phases: (1) Adaptation to the empty chamber, (2) Familiarity phase with two identical objects, and (3) Test phase (test day) for 2 consecutive days. On the 1st day, each animal was placed in an empty chamber for 10 min to become familiar with the new environment. On the 2nd day, first, two identical objects were placed diagonally in the two corners of the chamber, and then each animal was placed in the chamber for 10 min. On the same day, at an interval of 6 h, each animal was placed for 10 min in a chamber where one of the cubes was replaced with a new object, while the procedure was filmed with a camera.

During these 10 min, the animal searching time for the familiar and the new objects was measured and recorded with a timer. The exploratory behavior was defined as being within a 2 cm radius of the object and checking it by staring, smelling, and touching. The exploration ratio, as a time index for detecting a new object, was calculated by dividing the searching time for the new object by the total searching time for the familiar and new objects (Ganji et al., 2017). At the end of each test, the objects and device were cleaned with 70% ethanol to eliminate olfactory cues.

#### Barnes maze

The BM test also is used to measure spatial memory. This maze consists of a dark circular platform that is 120 cm in diameter with a height of 90 cm from the floor and consists of 20 holes (10 cm in diameter) that are located in a circle at the edge of the platform. The target hole of this system is connected to the escape box (10 × 10 × 15 cm^3^). The stimulus is a loud buzzer sound of 80 dB, which is 50 cm away from the device. All test sessions were recorded with a video camera. In the habituation session, the animals were given the opportunity to freely discover the device for 180 s before entering the escape box. Training sessions were held immediately after the habituation session for 3 days. If the rat did not reach the target hole, the experimenter directed the animal toward it at the end of the experiment. Animals remained in the escape box for 60 s. On the 4th day, spatial learning retrieval was assessed using a method similar to training experiments. Delay in reaching the target hole on training days, distance traveled, and the number of errors to reach the target area were recorded (Barnes, 1979; Kim et al., 2017).

#### Passive avoidance test

The PAT is a device to measure passive avoidance learning and memory, which is a rectangular box with dimensions of 20 × 20 × 40 cm and has two light and dark parts and a 60-watt lamp illuminates the light part. The two parts are connected by a hatch closed by a 7 × 9 cm guillotine door. At the bottom of the two parts, there are metal rods that are 1 mm in diameter and spaced 1 cm apart. An electric shock is applied through the rods by a stimulator to the animal’s feet in the dark part. This test is performed in three steps.

#### Adaptation stage

At the end of the treatment period, the animals were placed in the PAT. To make the animal familiar with the device, each animal was placed in the light part and after 30 s, the door was opened. According to the natural inclination to the dark environment, the animal entered the dark part. After closing the door and 30 s after being in the dark part, the animal was taken out and 30 min later, these steps were repeated.

#### Training procedure

To perform the learning test, 30 min after the second stage of adaptation, the animal was placed in the light compartment for 30 s, and then the guillotine door was opened. The delay in entering the dark part (step-through latency in the acquisition phase; STLa) was recorded for each animal at this stage, and then after the animal entered the dark part completely, the door was closed and a weak electric shock with an intense of 0.4 mA and frequency of 50 Hz was applied for 1.5 s through the metal rods. After 30 s, the animal was taken out from the dark part and this process was repeated after 2 min. When the animal avoided entering the dark part for 120 s, the learning test ended, otherwise, it received another shock after the animal entered the dark area again. Thus, the number of shocks that each animal received before avoiding entering the dark (the number of trials to acquisition) part was considered an indicator of the animal’s learning.

#### Retention test

To perform the memory test, 24 h after the learning test, the animal was placed in the light part and 30 s later, the guillotine door was opened and the animal’s behavior was evaluated for 300 s. During retention time, step-through latency (STLr) and time spent in the dark compartment (TDC) were measured and compared between groups.

#### Morris water maze

The MWM is used to assess spatial learning and memory in animal models (Rezvani-Kamran et al., 2017; Zarrinkalam et al., 2018). MWM consists of a circular pool (height: 65 cm, diameter: 185 cm) filled with water (25 ± 1°C) to a depth of 45 cm. The pool (painted black) is divided into four equal quarters with four starting points as east (E), west (W), north (N), and south (S). An escape route (an invisible platform 10 cm in diameter) is located 1.5 cm below the water level in the center of the northern quarter. The training phase was performed between 10:30 in the morning and 12:30 in the afternoon for 4 days, including two sections with four experiments. The rats of all groups were allowed to swim in the pool for 60 s from the starting points (E, W, N, and S) to reach the hidden platform. The rats remained on the platform for 30 s after being identified. In this method, an experimental interval of 5 min was also considered. A video camera above the tank connected to an exploration system recorded the requested parameters, including the escape delay to reach the hidden platform and the distance traveled by the swimmer. The platform was removed from the pool on day 5 and the animals swam for 1 min, and then the probe trial was done. In this stage, the elapsed time in the target quarter was recorded (Asadbegi et al., 2017; Zarrinkalam et al., 2016).

### Statistical analysis

Behavioral data from training phase of BM and MWM were analyzed by two-way analysis of variance (ANOVA) with repeated measures (fixe factors: treatment and within-subjects factors: day) followed by Tukey’s *post-hoc* test. Other behavioral data underwent one-way ANOVA. All data were tested for normal distribution with quantile-quantile (Q-Q) plot.

Statistical analysis was run in GraphPad Prism version 8.0 (GraphPad Software, San Diego, CA, United States). The results were expressed as mean ± the standard error of the mean (S.E.M.) and a *p*-value less than 0.05 was considered as the level of significance.

## Results

### OLZ can alleviate anxiety-like behavior following Aβ injection in the EPM test

**FIGURE 2:**
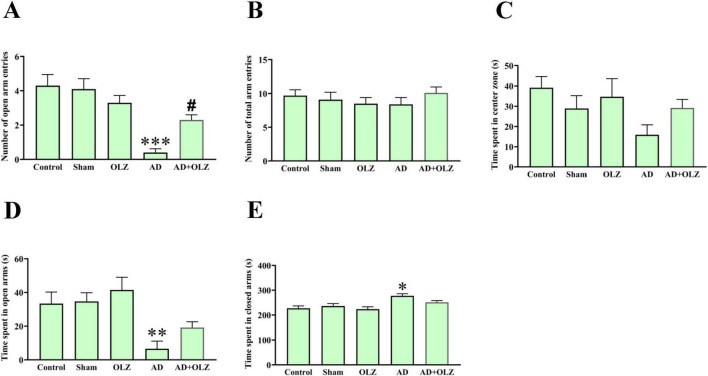
Anexity-like behavior was assessed using EPM. The number of open arm entries **(A)**, number of total arm entries **(B)**, time spent in central zone of maze **(C)**, time spent in open **(D)**, and closed arms **(E)**. All data are presented as mean ± SEM (one-way ANOVA, Tukey post-hoc test, *n* = 10). *p < 0.05, **p < 0.01, and ***p < 0.001 compared to the sham group. ^#^*p* < 0.05 compared to the AD group.

As shown in Figure 2B, there were no significant differences in the total number of arm entries (open + closed arms) among the groups (*p* > 0.05). However, the AD group exhibited a significant decrease in the number of open-arm entries compared to the sham group (*p* < 0.001, Figure 2A). Similarly, the time spent in the open arms was significantly less in the AD group compared to the sham group (*p* < 0.01, Figure 2D), indicating anxiety-like behavior in the AD group animals. The AD + OLZ group showed a significant increase in the number of open-arm entries compared to the AD group (*p* < 0.05).

A significant increase in the time spent in the closed arms was observed in the AD group compared to the sham group (*p* < 0.05, Figure 2E). There was no significant difference in the time spent in the closed arms between the AD + OLZ and sham groups (*p* > 0.05).

There were no significant differences in the time spent in the center zone of the EPM among the groups (*p* > 0.05, Figure 2C).

These results suggest that Aβ administration induces anxiety-like behavior in the AD model, as evidenced by reduced exploration of the open arms in the EPM test. OLZ treatment effectively alleviated this anxiety-like behavior, demonstrating its potential anxiolytic effects in Aβ-induced AD conditions.

### OLZ can restore cognitive performance following Aβ injection in the NOR test

**FIGURE 3:**
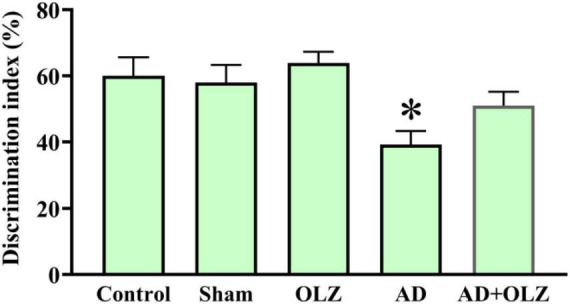
Recognition memory was assessed using NORT. Data are presented as mean ± SEM (one-way ANOVA, Tukey post-hoc test, *n* = 10). *p < 0.05 compared to the sham group.

The NORT was conducted to assess the impact of OLZ and AD on recognition memory performance. A one-way ANOVA indicated that the discrimination index was significantly lower in the AD group compared to the sham group (*p* < 0.001, Figure 3), suggesting recognition memory impairment in the AD rats. The AD + OLZ group exhibited a trend toward an increased discrimination index relative to the AD group.

These findings indicate that Aβ administration impairs recognition memory performance in AD rats, as demonstrated by a reduced discrimination index in the NORT. OLZ treatment showed potential to restore cognitive performance, highlighting its neuroprotective effects on recognition memory in the AD model.

### OLZ improves the memory of the rats in the BM test

**FIGURE 4:**
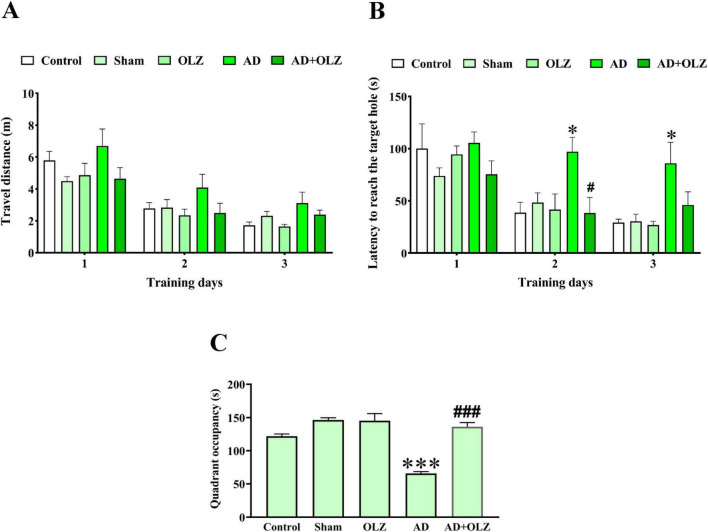
Spatial learning and memory were evaluated using BM. Traveled distance **(A)** and latency to reach the target hole **(B)** were analyzed by repeated measures two-way ANOVA. Quadrant occupancy **(C)** was analyzed using one-way ANOVA. All data are presented as mean ± SEM (*n* = 10). *p < 0.05 and ***p < 0.001 compared to the sham group. ^#^*p* < 0.05 and ^###^*p* < 0.001 compared to the AD group.

There were no significant differences among the experimental groups in the distance traveled to reach the target hole during the training days (*p* > 0.05, Figure 4A). However, the latency to reach the target hole was significantly increased in the AD group compared to the sham group on the 2nd and 3rd training days (*p* < 0.05 for each, Figure 4B). The AD + OLZ group showed a significant reduction in latency to reach the target hole compared to the AD group on the 2nd training day (*p* < 0.05).

On the probe day, the quadrant occupancy was significantly lower in the AD group compared to the sham group (*p* < 0.001). OLZ treatment significantly increased quadrant occupancy in the AD + OLZ group compared to the AD group (*p* < 0.001, Figure 4C).

These results suggest that AD impairs spatial memory and learning ability, as demonstrated by increased latency and reduced quadrant occupancy in the BM test. OLZ treatment effectively improved memory performance in AD rats, further supporting its therapeutic potential in mitigating cognitive deficits associated with AD.

### OLZ improves the passive avoidance learning and memory of the rats in the PA test

**FIGURE 5:**
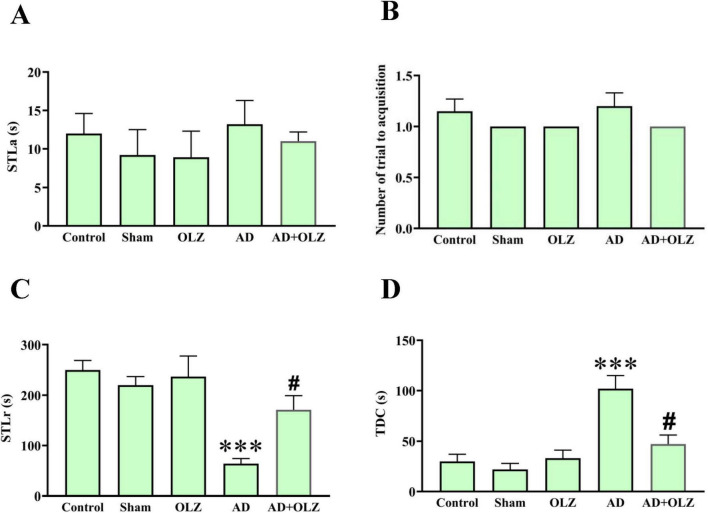
Passive avoidance memory was assessed using PAT. Step-through latency in the acquisition phase **(A)**, the number of trials to acquisition **(B)**, Step-through latency in the retention phase **(C)**, and time spent in the dark compartment **(D)**. were analyzed using one-way ANOVA. All data are presented as mean ± SEM (*n* = 10). ***p < 0.001 compared to the sham group. ^#^*p* < 0.05 compared to the AD group.

During the adaptation phase, no significant differences in STLa were observed among the experimental groups (*p* > 0.05, Figure 5A). Similarly, there were no significant differences in the number of acquisition trials among the groups (*p* > 0.05, Figure 5B), although the AD group showed a trend toward an increased number of acquisition trials compared to the sham group.

In the retention phase, the AD group demonstrated a reduced STLr compared to the sham group (*p* < 0.001, Figure 5C). The AD animals treated with OLZ (the AD + OLZ group) showed an STLr similar to the sham group, which was higher than that of the AD group (*p* < 0.05). A significant increase in the TDC was observed in the AD group compared to the sham group (*p* < 0.001, Figure 5D). Treatment with OLZ significantly decreased the TDC in the AD + OLZ group compared to the AD group (*p* < 0.05).

These results indicate that Aβ administration impairs learning and memory retention in the PA test, as evidenced by reduced STLr and increased TDC. OLZ treatment effectively improved both retention latency and memory performance, suggesting its ability to mitigate learning and memory deficits associated with AD.

### OLZ improves the spatial memory of the rats in the MWM test

**FIGURE 6:**
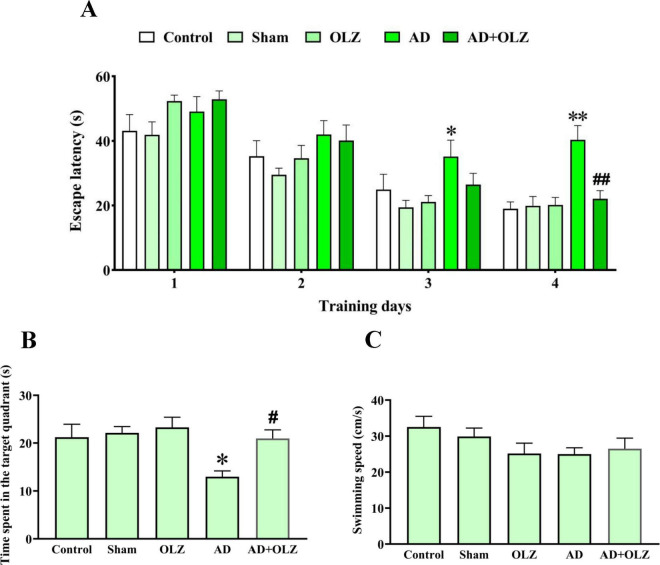
Spatial cognition performance was assessed using MWM. Escape latency during the training days **(A)** was analyzed by repeated measures two-way ANOVA. Time spent in the target quadrant **(B)** and swimming speed in the probe day **(C)** were analyzed by one-way ANOVA. All data are presented as mean ± SEM (*n* = 10). *p < 0.05 and **p < 0.01 compared to the sham group. ^#^*p* < 0.05 and ^##^*p* < 0.01 compared to the AD group.

The escape latency, which measures the time taken by a rat to locate the hidden platform during training, was assessed during the training days (Figure 6A). No significant differences in escape latency were observed on days 1 and 2 among the experimental groups (*p* > 0.05). However, the AD group exhibited significantly higher escape latency compared to the sham group on training days 3 (*p* < 0.05) and four (*p* < 0.01). Treatment with OLZ reduced the escape latency on the 4th day in the AD + OLZ group compared to the AD group (*p* < 0.01).

During the probe trial, the time spent in the target quadrant and swimming speed were analyzed. The AD group spent significantly less time in the target quadrant compared to the sham group (*p* < 0.05). The AD + OLZ group spent more time in the target quadrant than the AD group (*p* < 0.05, Figure 6B).

There were no significant differences in swimming speed among the experimental groups (*p* > 0.05, Figure 6C), indicating that the reduced time spent in the target quadrant by the AD group was not due to locomotor deficits.

These findings demonstrate that Aβ administration impairs spatial learning and memory, as shown by increased escape latency and reduced time spent in the target quadrant. OLZ treatment effectively improved spatial memory in the AD rats, reinforcing its neuroprotective and therapeutic potential in mitigating AD-related cognitive deficits.

## Discussion

In the present study, the following results were obtained: ICV injection of Aβ to create the AD model impaired memory in behavioral tests, including NOR, MWM, BM, PAL, and EPM. Taking OLZ in the AD group improved the memory and learning impairment in behavioral tests. The results of this study showed that treatment with OLZ can somewhat reduce the detrimental effects of Aβ injection and AD induction on learning and memory. In the following paragraphs, we discussed the different effects of OLZ on various aspects of memory and learning, as well as the cellular and molecular mechanisms of the effect of this compound on the nervous system.

Consistent with the findings of this study, it was found that OLZ improved the spatial memory function of mice in the MWM (Hou et al., 2006). OLZ significantly reduced the time to reach the target quadrant and decreased working memory errors in the MWM. These results indicate that OLZ can improve cognitive function and memory (Mahmoud et al., 2019). OLZ improved memory in rats exposed to stress before birth (Nowakowska et al., 2014). The results of another study showed that OLZ has a protective effect against MK-801-induced cognitive impairment in mice through stimulating neurogenesis (Song et al., 2016). OLZ has been reported to reduce phencyclidine-induced acute reverse learning disorder (McLean et al., 2010). In addition, OLZ has been shown to significantly increase the time spent in the target quadrant of the MWM test in mice exposed to unpredictable chronic mild stress (UCMS) (Gumuslu et al., 2013).

OLZ alleviated dopamine D2 receptor priming and cognitive impairment and reduced neurotrophins and acetylcholinergic markers produced by D2 receptor priming in the hippocampus (Thacker et al., 2006). OLZ may have protective effects on procedural learning (even doses, at which D2 striatal receptors are similar to HAL). This protective effect of OLZ may be related to its atypical pharmacological properties (Paquet et al., 2004). Treatment with OLZ in adulthood significantly increased ChAT RNA expression in the cerebellum. OLZ reduced cognitive deficits induced by quinpirole treatment in infancy (Brown et al., 2008). In patients with memory impairment, OLZ is more desirable than antipsychotic HAL, especially in patients with cholinergic deficiency (Brown et al., 2008). Regarding extrapyramidal syndromes, the advantages of OLZ over HAL and risperidone can be extended to its superiority in procedural learning (Purdon et al., 2003).

Electrophysiological results confirm the effects of OLZ on memory and learning. In this regard, treatment with OLZ increased the electrophysiological I/O response of CA1 pyramidal cells and synaptic transmission (Shim et al., 2012). The results of another electrophysiological study revealed a new mechanism of OLZ and showed that the anticholinergic properties of OLZ affect glutamate signaling and synaptic plasticity (Song et al., 2017).

On the other hand, the results of another research indicated the destructive effects of OLZ on memory and learning. OLZ impaired cognitive function in naive animals (Mutlu et al., 2011). The results of another study showed that OLZ significantly increased the average delay in reaching the platform and decreased the number of crossings in the target area (Ning et al., 2017). Long-term oral treatment with OLZ may impair the cognition function components involved in schizophrenia (Terry et al., 2008). OLZ also impaired spatial reversal learning in mice in the MWM (Song et al., 2017). In another study, prenatal administration of OLZ did not impair learning or memory retention (Rosengarten and Quartermain, 2002). According to another study, OLZ appears to be less involved in inattentive learning and movement speed than classical neuroleptics (Stevens et al., 2002).

We can consider the neuroprotective role of OLZ as one of the reasons for the positive effect of this compound on memory and learning. Consistent with our findings, it has been reported that a high dose of OLZ improved hippocampal nerve damage due to the administration of kainic acid. These results indicate that OLZ has neuroprotective effects in rat models with nerve damage (71). OLZ (0.1 mg/kg) has been reported to have significant neuroprotective effects in mice after focal ischemia-induced brain injury (Yulug et al., 2008). However, it has been shown that the side effects of OLZ at high doses may limit its neuroprotective effect, whereas, at low doses, it has been reported to have a neuroprotective effect (Yulug and Kilic, 2009). Chronic pretreatment with OLZ, effectively reduced 24-h methamphetamine (METH)-induced mortality, and tyrosine hydroxylase levels in caudate-putamen (CPu) significantly improved. In addition, it has been reported that the neuroprotective properties of OLZ may be associated with its mitigating effects on METH-induced hyperthermia and its preventive role in reducing Bcl-2 (an anti-apoptotic gene product) induced by METH in the CPu. These results suggest that OLZ may be a neuroprotective agent and this property of OLZ may contribute to its therapeutic effects in the treatment of schizophrenia (He et al., 2004).

An immunohistochemical study using 50-bromodeoxyuridine showed a positive association between OLZ sensitivity (e.g., changes in avoidance suppression induced by OLZ during the day) and hippocampal cell proliferation (Chou et al., 2015). In another study, the number of retinal ganglion cells (RGCs) was significantly higher in the OLZ -receiving group than in the control group. OLZ protects RGC from NMDA-induced damage; thus, OLZ may have potential neuroprotective effects (Onder et al., 2013). In neuronal cultures, OLZ protected neurons from damage caused by thapsigargin. OLZ treatment increased repressed neuronal differentiation due to exposure to thapsigargin (Kurosawa et al., 2007). OLZ treatment also increased the phosphorylation of AMPK in PC12 cells. It has been shown that pretreatment with OLZ can protect PC12 cells from rotenone-induced apoptosis. In addition, pretreatment with OLZ can suppress rotenone-induced depolarization in mitochondria and consequently, protect cells (Xiong et al., 2020). According to another study, chronic administration of OLZ (21 days) increased the number of newborn cells in the hippocampal dentate gyrus by the same amount of fluoxetine. The results of the mentioned study showed that antidepressants or atypical antipsychotics can increase glia proliferation in limbic brain structures (Kodama et al., 2004). OLZ treatment prevented the destructive effects of phencyclidine on normal neurons (but not on the neurons of mice, in which NRG1 was knocked out). These results suggest that NRG1 mediates the prophylactic effects of OLZ on neurological dysfunction and the expression of phencyclidine-induced synaptic proteins (Zhang et al., 2016). Another study showed that positive OLZ effects on the improvement of ER stress may be due to the cellular mechanisms of atypical antipsychotics in the protection of neurons and NSCs (Kurosawa et al., 2007). OLZ may also have neuroprotective effects through erythropoietin expression (Pillai and Mahadik, 2006).

We believe that some of the positive effects of OLZ on memory and learning may be due to the effect of this compound on BDNF levels. In support of this hypothesis, OLZ administration for 5 weeks significantly increased BDNF levels in the cortex and hippocampus (Czubak et al., 2009). The results of another study showed that the increase in serum BDNF in OLZ monotherapy is significantly higher than lurasidone (Jena et al., 2019). The results of another study showed that treatment with OLZ increased the levels of Akt, CREB, BDNF, Bcl-2, and BAD in the prefrontal cortex, hippocampus, and striatum (Réus et al., 2012). Also, administration of OLZ for 1 week restored Bcl-2 expression to pre-stress levels, and administration for 3 weeks caused overexpression of BDNF in hippocampal neurons (Luo et al., 2004). Another study showed that 4 weeks of OLZ treatment increased Bcl-2 and CREB levels in the dentate gyrus and CA1 region of the hippocampus. Four weeks of OLZ treatment regulated upward BDNF in the dentate gyrus. These results suggest that upregulation in Bcl-2 and CREB may underlie OLZ neuroplastic action (Hammonds and Shim, 2009). The results of another study showed that upregulation of OLZ on BDNF gene transcription is associated with increased CREB transcription through the PKA, PI3K, PKC, and CaMKII signaling pathways and OLZ may exert neuroprotective effects through these signaling pathways in neurons (Lee et al., 2010). OLZ at the doses of 1 and 10 mg/kg improved a decrease in the levels of BDNF, ERK, and CREB by SRS in PTSD animals. In addition, it prevented an increase in Caspase-3, which is a downstream apoptotic agent, at these doses (Reddy and Krishnamurthy, 2018). Real-time PCR results showed that the expression levels of CREB and BDNF genes significantly reduced in the group exposed to mild chronic unpredictable stress.

OLZ has antioxidant effects. We suspect that in our study, this compound also affected memory and learning due to its antioxidant properties. Antioxidants have been shown to have positive effects on memory and learning. Treatment with OLZ for 2 months showed a significant change in serum TAC (37.8%) and serum MDA levels (22.2%) compared to pre-treatment values. These data suggest that treatment with OLZ for 2 months at least partially improved the adverse effects on the antioxidant defense mechanism of schizophrenic patients (Al-Chalabi et al., 2009). Also, MDA levels showed a significant decrease in the OLZ treatment group compared to the control group (Onder et al., 2013).

Anxiety is a prevalent non-cognitive symptom of AD, often correlating with neurodegeneration, disease progression, and increased levels of stress biomarkers. In our study, the EPM test was employed to assess anxiety-like behaviors in experimental rats. The results showed that animals in the AD group spent significantly less time in the open arms and exhibited fewer open-arm entries compared to the sham group, indicating heightened anxiety-like behavior. However, the AD + OLZ group demonstrated a significant increase in both the time spent in the open arms and the number of open-arm entries, suggesting that OLZ treatment alleviated anxiety in AD-induced animals.

Anxiety in AD models has been extensively investigated, with studies highlighting its association with oxidative stress, neuronal death, and Aβ toxicity. For instance, research has demonstrated that Aβ1-42 increases anxiety-like behavior through mechanisms involving oxidative damage and plaque accumulation (Tork et al., 2024). The anxiolytic effects observed in our study align with OLZ’s known pharmacological properties, including its ability to counteract oxidative stress and neurotoxicity induced by Aβ. This is consistent with findings from other studies, such as those showing L-carnitine alleviating anxiety in Aβ-induced AD models by improving oxidative-antioxidant balance (Tork et al., 2024).

OLZ, an atypical antipsychotic, possesses inherent anxiolytic properties that are independent of its antipsychotic effects. Preclinical evidence suggests that OLZ reduces anxiety in various behavioral models, including stress-induced anxiety-like behavior tests (Locchi et al., 2008). In clinical settings, OLZ has shown efficacy in reducing anxiety symptoms in patients with bipolar disorder (Maina et al., 2008), social anxiety disorder (SAD; Moretti et al., 2004), and vascular dementia (Barnett et al., 2002). Furthermore, OLZ has been reported to significantly improve anxiety-related symptoms in AD patients, as demonstrated by reductions in Neuropsychiatric Instrument (NPI) anxiety scores compared to placebo (Mintzer et al., 2001).

It is important to note that OLZ’s anxiolytic effects can vary based on sex and treatment duration. Chronic treatment with OLZ has been shown to decrease anxiety in male rats but increase it in females under specific conditions (Lockington and Hughes, 2021). Despite these nuances, the overall anxiolytic potential of OLZ is supported by substantial preclinical and clinical evidence, making it a promising therapeutic agent for addressing anxiety symptoms in AD.

As a result, our study corroborates the anxiolytic effects of OLZ in an Aβ-induced AD model, highlighting its dual role in cognitive protection and anxiety alleviation. These findings underscore the potential of OLZ as a viable pharmacological option for managing anxiety in AD, which is a critical component of the disease’s non-cognitive symptomatology.

## Conclusion

In summary, this experiment showed that ICV injection of Aβ to create the AD model reduced memory in behavioral tests, including NOR, MWM, BM, PAL, and EPM, whereas, OLZ improved the performance of memory and learning in behavioral tests in the Aβ-induced AD model rats. In general, according to the results of this study and other studies discussed, OLZ can be considered as a suitable drug in terms of prophylaxis due to its functional mechanisms (neuroprotective, antioxidant, anti-inflammatory, and synaptic plasticity enhancer properties). Thus, it can be considered a treatment for AD.

## Data Availability

The original contributions presented in the study are included in the article/supplementary material, further inquiries can be directed to the corresponding author.
